# Converging Currents in Climate-Relevant Conservation: Water, Infrastructure, and Institutions

**DOI:** 10.1371/journal.pbio.1001159

**Published:** 2011-09-06

**Authors:** John H. Matthews, Bart A.J. Wickel, Sarah Freeman

**Affiliations:** 1 Global Initiatives Division, Conservation International, Arlington, Virginia, United States of America; 2 Conservation Science Program, World Wildlife Fund, Washington, D.C., United States of America

## Abstract

Ecologists and economists have long talked past each other, but climate change presents similar threats to both groups. Water may serve as the best means of finding a common cause and building a new vision of ecological and economic sustainability, especially in the developing world.

During a 2008 panel for the Intergovernmental Panel on Climate Change's launch of a report on water and climate [1], a hydrologist and an engineer called for additional monitoring and research to detect and attribute the effects of anthropogenic climate change. The third member of the panel, a frustrated World Bank infrastructure lender, declared in response, “I can't wait thirty years for precise science to tell me how much global warming contributed to a particular drought or flood. I want to see climate adaptation programs based on non-precise decision making. I need to make investment decisions now.”

In theory, this lender should be finding guidance from conservation science, particularly ecology. Sustainable management of natural resources such as forests, soils, water, and fisheries are at the heart of conservation, and these resources are the building blocks for “green” cities, energy production, agriculture, and water supply and sanitation systems. Relatively stable ecosystems and species dynamics are indicative of sustainable resource use, and conservation science has been broadening this knowledge to buffer ecosystems and species from negative climate change impacts [2]–[5]. Unfortunately, the means of applying this new literature to sustainable economic development remains unclear. Conservation scientists and economic investors continue to work largely in isolation from one another. Poor investment and management decisions risk climate-initiated ecological transformations, which in turn could trigger economic crises. In 2004, for instance, Rwanda was briefly threatened with the loss of 90 percent of its electrical capacity following long-term wetlands destruction and a drought [6], putting the country's development trajectory at risk and worsening the ecological damage already incurred.

The difficulty of determining future hydrological conditions based on past records of climate and hydrologic regimes has been the “death of stationarity” [7], which refers to the assumption that future climate conditions have “predictable uncertainty”; that is, the frequency and severity of flood or drought events can be accurately estimated, allowing water managers (and economists) to manage risk. Climate change undermines this assumption by suggesting that the future holds unpredictable uncertainty. The water resource management community has not yet developed an alternative vision capable of embracing this new reality [8].

Not all indications are bad, however. One of the most promising combinations of conservation science with a more resilient vision of ecological sustainability are infrastructure design and operations, which is also a growing focus for mainstream economic decision makers. Recent reports from the United Kingdom and United States governments and the World Bank highlight the critical need to modify how we plan and finance infrastructure to become more climate-adaptive, particularly water infrastructure [9]–[11].

Water resources management and water infrastructure mediate much of our influence on both terrestrial and freshwater ecosystems. The development of complex human societies is also a history of our increasing mastery of freshwater resources. Wetlands management for rice cultivation dates back about 8,000 years BP in eastern China [12]. Some still-operational dams in Yemen and Turkey date back more than 2,000 years. More than 40,000 large dams exist in the United States alone, most built between 1950 and 1970 [13]. The rise of China, India, and Brazil marks a new “golden era” of rapid water infrastructure development [14]. Economic development for many governments has come to mean the sustained (if not sustainable) mastery of freshwater resources, typically in service of agriculture, energy, and urban and industrial needs.

Climatic change and variability has always presented serious challenges to water resources management. The hydrological cycle—surface and ground water, precipitation, soil moisture, snowpack and glaciers, flow regime and hydroperiod, runoff, and evapotranspiration—responds to even small shifts in climate in often unpredictable ways [1]. Archaeological reconstructions show that humans have often had to react dramatically to climate regime shifts through the medium of water infrastructure in order to survive, often failing in the process [15]. According to the United Nations World Water Assessment Programme, for humans, climate change is water change [16].

From a social perspective, the challenges presented by climate change impacts are especially acute today in a globalizing world. The impacts of too much and too little water extend far beyond the localities where they occur and are capable of disrupting national food and energy security as well as the global economy [17],[18]. For instance, the 2008 intensification of the Australian Murray-Darling drought contributed to the rapid rise in food prices in India [19], while in January 2011 China committed almost US$600 billion to more sustainable water management, especially for its drying north [20].

Combining measures of ecosystem integrity, species health, and socioeconomic indicators to evaluate water resource management and climate change adaptation represents a paradigm shift for both sustainable development and conservation science. However, our ability to project future eco-hydrological conditions with confidence is severely limited due to the deep uncertainties in climate models, particularly for precipitation [1], and many ecologists struggle to find actionable relevance in these models at the local “project” or “site” scale where most policy and resource management decisions reside [21]. Hydropower, irrigation, large-scale water diversions, flood control levees and bypass structures, storage systems, wells and boreholes, and treatment and sanitation facilities are traditionally designed to function over decadal or even century-scale time spans. Traditional methods for infrastructure design that do not take account of a non-stationary climate over their operational lifetime render infrastructure vulnerable to climate shifts. Climate change represents a serious impediment for water resource management and economic development success [22]–[24].

Long-functioning dams face new operational hurdles that demonstrate the consequences of stationarity assumptions. Hoover Dam in the Colorado River basin, for instance, was designed in the 1930s based on observations made during three of the wettest decades of the past millenium, but Lake Mead now consistently stores only about 30 percent of its designed capacity. Precipitation trends are now moving closer to the 1,200-year mean [25], which implies that the existing infrastructure and patterns of water allocation throughout the basin face difficult tradeoffs between agriculture, cities, and energy production. Notably, the winter of 2010–11's elevated precipitation levels have not prompted water managers to declare the current “drought” over. Water infrastructure in regions where the climate is shifting quickly, such as the Himalayas or the Andes [26],[27], are designed with an assumption of stationarity, but many are likely to already be facing mismatches with their ambient climate.

As a result, the potential impacts on infrastructure investments are massive. A group of development banks calculated that most developing countries spend 2 to 6 percent GDP on infrastructure development, amounting to almost half of all international financial institutions' lending [28]. The Organization for Economic Co-operation and Development has estimated that about 40 percent of all development investments are at risk due to climate change [29]. The Secretariat for the Convention on Biological Diversity projects that public funding of infrastructure by 2020 will total US$400 billion in Asia and US$10 billion in Africa [30]. Private investment sources, aid agencies, and development banks together build a global water sector measuring in the tens of billions annually [16]. World Bank investments in water alone totaled over US$10 billion in fiscal year 2010 [11].

Climate-infrastructure mismatches are likely to produce a host of serious repercussions for ecosystems and economies. In the developing world, climate-infrastructure mismatches are likely to reduce economic growth through low rates of electrical production or irregular agricultural harvests while wasting scarce investment capital. Ecologically poorly designed water infrastructure is likely to reduce the inherent resilience and adaptive capacity of these nations' ecosystems, permanently altering lakes, rivers, soils, and fisheries. Low levels of water security and high biodiversity threats show a close correlation globally [31]. Climate-infrastructure mismatches may actually make poor nations even poorer.

How can conservation science provide the practical decision making tools for funding, designing, and operating water infrastructure that enables both economic and ecological sustainability? We propose a recursive three-step process (Figure 1):

- Consider alternatives to building new infrastructure. Particularly for large infrastructure projects such as Hoover Dam in the US or China's South-to-North Water Transfer Project, the risks for investors, communities, and ecosystems are extremely high given uncertainties in future hydrological conditions. One or two decades of apparently successful implementation are likely to be followed by very difficult tradeoffs between socioeconomic and ecological impacts, with both likely losing to the overly optimistic expectations created when long-term impacts and non-stationarity are not considered. More effective approaches to reduce ecosystem impacts and maximize operational lifetimes include reducing water and power demands, building infrastructure in stages as climate trends become more clear, or adjusting agriculture irrigation schemes and crop selections to cope with more variable water supplies.
- Explicitly integrate ecosystems into infrastructure development. The emerging practice of integrating functional and intact ecosystems into water resource management can help humans and ecosystems adjust to emerging climate impacts, an approach to climate change adaptation referred to in The Netherlands as “building with nature” [32]. Focusing on seasonal flow management systems [11],[33], “making room for the river” by restoring floodplain dynamics [34], and development and/or redevelopment of decision trees for policymakers considering “bioshields” for extreme weather events [35] are all powerful new tools and approaches that link the sustenance and viability of both social and ecological systems within a sound conservation framework.
- Reduce the vulnerability of the infrastructure and its impacted ecosystems over the operational lifetime of the project. The assumption of stationarity results in infrastructure designed for a single climate future, but designing for multiple potential climate regimes may be a more conservative and economically viable alternative [11]. Articulating additional future climate scenarios bridges the scientific discussion of uncertainty with the engineering and investment concerns about operational and investment risks. Equally important with infrastructure design is the need to create institutional structures capable of integrating monitoring data into flexible infrastructure operations [36], as well as more research into bio-indicators to track ecological integrity through time and anticipate ecological tipping points. This will allow emerging conditions to be anticipated, operational efficiency maintained, and ecological impacts reduced [37],[38]. The development of a dynamic seasonal river flow program in Tanzania's Pangani basin by the International Union for Conservation of Nature (IUCN), for instance, represents an exciting new approach to balance multiple allocation demands, economic growth, and probable long-term declines in water availability while reducing human-induced pressures on freshwater ecosystems; meanwhile, Rwanda has reduced its dependency on hydropower following the 2004 drought and undertaken extensive wetlands restoration work to stabilize hydrologic regimes [39]. The impacts of existing infrastructure should also be reconsidered. Processes for infrastructure evaluation and relicensing and land-use classification schemes (such as redefining a “100-year flood event”) provide opportunities to engage with the finance sector to incentivize the reduction of long-term vulnerabilities to humans and ecosystems.

**Figure 1:**
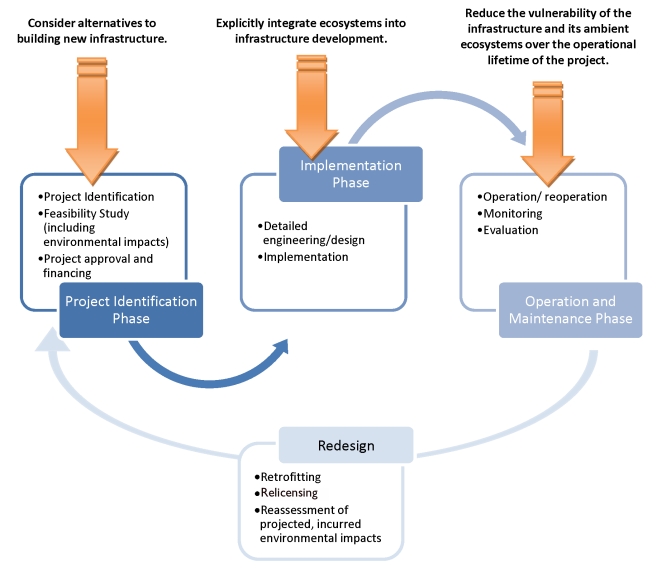
Proposed actions for how conservation science can improve water infrastructure. Note that the orange arrows represent the potential points for engagement in the infrastructure development process.

Economic development must occur, but climate change is making sustainable development a much broader and more plastic concept that is harder to define and achieve. Conservation science should play an essential role in facilitating the transition to more flexible approaches to natural resource management by focusing on the issues that deeply concern policymakers and profoundly modify ecosystems, especially over long time scales. Climate-sustainable water resource management should be part of the long-term strategy of the conservation community to help economies and terrestrial and freshwater ecosystems to adjust to an uncertain future. Given the risks for human communities and ecosystems from climate change, ecologists working in the developing world need to think more like development economists, and economists need to think more like ecologists.
